# Mitochondria-Targeted Lipid Nanoparticles Loaded with Rotenone as a New Approach for the Treatment of Oncological Diseases

**DOI:** 10.3390/molecules28207229

**Published:** 2023-10-23

**Authors:** Leysan Vasileva, Gulnara Gaynanova, Darya Kuznetsova, Farida Valeeva, Anna Lyubina, Syumbelya Amerhanova, Alexandra Voloshina, Guzel Sibgatullina, Dmitry Samigullin, Konstantin Petrov, Lucia Zakharova

**Affiliations:** 1 Arbuzov Institute of Organic and Physical Chemistry, FRC Kazan Scientific Center, Russian Academy of Sciences, 8 Arbuzov Str., Kazan 420088, Russia; 2 Kazan Institute of Biochemistry and Biophysics, FRC Kazan Scientific Center, Russian Academy of Sciences, 2/31 Lobachevsky Str., Kazan 420111, Russia

**Keywords:** mitochondria, cationic surfactant, triphenylphosphonium, imidazolium, liposome, rotenone, cancer, colocalization

## Abstract

This research is based on the concept that mitochondria are a promising target for anticancer therapy, including thatassociated with the use of oxidative phosphorylation blockers (mitochondrial poisons). Liposomes based on L-α-phosphatidylcholine (PC) and cholesterol (Chol) modified with cationic surfactants with triphenylphosphonium (TPPB-n, where n = 10, 12, 14, and 16) and imidazolium (IA-n(OH), where n = 10, 12, 14, and 16) head groups were obtained. The physicochemical characteristics of liposomes at different surfactant/lipid molar ratios were determined by dynamic/electrophoretic light scattering, transmission electron microscopy, and spectrophotometry. The hydrodynamic diameter of all the systems was within 120 nm with a polydispersity index of no more than 0.24 even after 2 months of storage. It was shown that cationization of liposomes leads to an increase in the internalization of nanocontainers in pancreatic carcinoma (PANC-1) and duodenal adenocarcinoma (HuTu 80) cells compared with unmodified liposomes. Also, using confocal microscopy, it was shown that liposomes modified with TPPB-14 and IA-14(OH) statistically better colocalize with the mitochondria of tumor cells compared with unmodified ones. At the next stage, the mitochondrial poison rotenone (ROT) was loaded into cationic liposomes. It was shown that the optimal loading concentration of ROT is 0.1 mg/mL. The Korsmeyer–Peppas and Higuchi kinetic models were used to describe the release mechanism of ROT from liposomes in vitro. A significant reduction in the IC_50_ value for the modified liposomes compared with free ROT was shown and, importantly, a higher degree of selectivity for the HuTu 80 cell line compared with the normal cells (SI value is 307 and 113 for PC/Chol/TPPB-14/ROT and PC/Chol/IA-14(OH)/ROT, respectively) occurred. It was shown that the treatment of HuTu 80 cells with ROT-loaded cationic liposomal formulations leads to a dose-dependent decrease in the mitochondrial membrane potential.

## 1. Introduction

The critical role of mitochondria in the functioning of healthy and transformed cells makes these organelles an ideal target for pharmaceutical substances for the treatment of various pathologies (for instance, neurodegenerative, oncological, and cardiovascular diseases) [1,2]. Cellular processes such as energy production, calcium homeostasis, production of reactive oxygen species, cell survival, apoptosis, and regulation of the immune response are largely controlled by the cell mitochondria [3,4]. Mitochondrial medicine is a field of science and practice that deals with mitochondrial dysfunction in one or more of the listed cellular processes leading to disease progression [5,6,7,8,9]. Cancer development is closely related to the proliferation of transformed cells. A hallmark of tumor cell metabolism is increased glucose uptake and the enzymatic conversion of glucose to lactate even in the presence of oxygen (the Warburg effect) [10]. Aerobic glycolysis in cancer cells was long believed to be the result of mitochondrial dysfunction, which in turn was thought to be the cause of cancer. However, later it was proved that the Warburg effect is not the cause of malignant transformation but its consequence and adaptation to hypoxia in tumor cells [11,12]. New therapeutic methods should take into account the main factors affecting the resistance of tumors to chemotherapy. Cell metabolism-focused research offers new perspectives that may contribute to the development of innovative and effective medicines [13,14].

There are strategies for targeting mitochondria, such as the use of mitochondrial leader sequences, mitochondrial peptides, delocalized cations, and self-assembled bolaform dequalinium structures (DQAsomes) [1,15]. Among them, the use of delocalized cations has attracted attention due to our experience in cationic surfactant research [16,17,18]. In the case of delocalized cations, the mitochondrial targeting strategy is based on the high negative charge of the mitochondrial membrane (about −180 mV) and gentle passage of lipophilic cations through the cell membrane, maintaining its integrity. Mitotropic cations, for instance, alkyltriphenylphosphonium cations, rhodamine, and cyanine cations, could be covalently attached to the functional moiety through a linker [15]. For instance, conjugates of rhodamine B with pentacyclic triterpenoic acids (oleanolic, ursolic, betulinic, platanic, and asiatic acids) [19], with hydroxy core-modified porphyrin [20], and with zinc complexes [21] have been obtained and investigated. A variety of work on delocalized cations was carried out on the triphenylphosphonium (TPP) cation, which was covalently linked to doxorubicin [22], glycyrrhetinic acid [23], betulin and betulinic acid [24], ginsenosides (the main active components in ginseng) [25], etc. There are examples of TPP cation introduction into a polymer matrix: the polyethylene glycol-phosphatidylethanolamine conjugate with the TPP group and paclitaxel demonstrated enhanced cytotoxicity and anti-tumor efficacy [26]; methoxy polyethylene glycol—TPP conjugate with biodegradable linkage and two disulfide bonds could cause rapid doxorubicin release with enhanced mitochondrial uptake [27]; and D-α-tocopheryl polyethylene glycol 1000 succinate—TPP conjugate was incorporated into liposomes [28]. However, covalent modification can potentially affect activity. Therefore, as an alternative, the noncovalent modification of nanocontainers can be used [29]. Modified liposomes can also be obtained by introducing a TPP group into phospholipids [30,31,32] or by the noncovalent modification of nanoparticles with TPP-containing amphiphilic derivatives [33,34,35,36,37]. The latter is achieved by a hydrophobic tail that anchors into the lipid bilayer, while TPP is located on the inner and outer liposome surface. Previously, for the first time, we have used a homologous series of alkyltriphenylphosphonium [35] and 1-methyl-3-alkylimidazolium [38] bromides for the noncovalent modification of liposomes. It has been established that they are able to colocalize with mitochondria [34,35].

A modern approach in the treatment of oncological diseases resistant to traditional types of chemotherapy is the use of oxidative phosphorylation blockers. Currently, more than 20 compounds are known to directly act on mitochondria and cause the death of unhealthy cells (such as rotenone (ROT), antimycin A, oligomycin, etc.) [39]. Therefore, studies with surfactant-modified liposomes with a delocalized charge were developed by loading them with mitochondrial poison.

**Figure 1:**
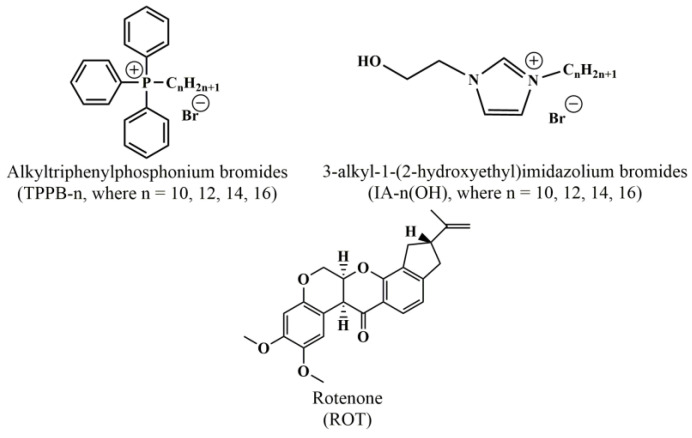
Structural formulas of the investigated compounds.

In the framework of this research, liposomal formulations based on L-α-phosphatidylcholine (PC), cholesterol (Chol), and two types of surfactants with a delocalized positive charge, namely, n-alkyltriphenylphosphonium bromides (TPPB-n, where n = 10, 12, 14, and 16) and 3-alkyl-1-(2-hydroxyethyl)imidazolium bromides (IA-n(OH), where n = 10, 12, 14, and 16) (Figure 1), were created. The obtained modified liposomes were loaded with the mitochondrial poison ROT. After the optimization of the liposomal formulation, in vitro studies on the colocalization of modified liposomes with the mitochondria of duodenal adenocarcinoma (HuTu 80) and pancreatic carcinoma (PANC-1) cells were carried out. A comparative analysis of all the results obtained for the traditionally used triphenylphosphonium cation and the new, from the point of view of mitochondrial targeting, imidazolium cation was carried out. In both cases, the modification of the lipid bilayer was carried out by the method of noncovalent incorporation of amphiphilic compounds, which is based on thin lipid film hydration.

## 2. Results and Discussion

Previously developed formulations based on 1,2-dipalmitoyl-sn-glycero-3-phosphocholine (DPPC) and cationic surfactants [34,35] were optimized by replacing DPPC with soy L-α-phosphatidylcholine (PC) and cholesterol (Chol). It is known that liposomes based on lipids with unsaturated bonds in the hydrophobic tails (for example, soy PC) are characterized by high membrane permeability and low stability [40]. Such disadvantages of liposomal systems can be prevented by adding Chol to the liposome composition by improving the packaging of phospholipids and affecting the rigidity and fluidity of the lipid bilayer [41], thereby leading to an increase in the stability of liposomes [42]. The formulation requires careful selection of the component ratio, so the lipid/surfactant molar ratio was varied over a wide range, namely, 50/1, 35/1, and 25/1. Because size and electrokinetic potential are of significant importance in creating nanoscale delivery systems, the first step involved measuring the physicochemical characteristics of liposomes, namely, the hydrodynamic diameter (D_h_), polydispersity index (PdI), and zeta potential (ζ), using dynamic and electrophoretic light scattering (DLS/ELS). The hydrodynamic diameter of the modified liposomes was approximately 100–120 nm with a PdI of no more than 0.24 (Table 1). It is worth noting that the modification of the liposomes with cationic surfactants led to a slight compaction and reduction in size compared with the unmodified liposomes. According to the monitoring of the liposome stability over time, the systems remained stable for more than two months. During the storage period, the liposomal formulations maintained a high degree of monodispersity (the PdI was less than 0.27) and a size of no more than 134 nm (Table 1).

Because the main task of the modification of the liposomes with surfactants was the cationization of the liposome surface, special attention was devoted to measuring the zeta potential of the nanoparticles. As shown in Figure S1a,b, increasing the hydrophobicity of TPPB-n and IA-n(OH) led to an increase in the positive charge of the liposomes. For instance, in the series of liposomes modified with IA-n(OH), the zeta potential value increased for the dodecyl, tetradecyl, and hexadecyl homologues in lines +26 mV, +39 mV, and +44.2 mV, respectively. Such an effect of the surfactant tail is reliably documented both by our research group and by others [35,43,44,45]. For the tetradecyl homologue, a sufficient zeta potential was achieved. It is interesting that the zeta potential of the liposomes modified with IA-n(OH) increased smoothly depending on the surfactant hydrocarbon tail length, while the values for the liposomes with TPPB-n changed insignificantly (Figure S1a,b). Additionally, increasing the concentration of TPPB-n and IA-n(OH) in the lipid bilayer contributed to a slight increase in the liposome zeta potential (Figure S1c,d). After 2 months of storage, the zeta potential of the IA-n(OH) liposomes decreased, while the zeta potential of the TPPB-n liposomes, on the contrary, increased, which was also shown earlier [36]. In addition, for the liposomes modified with a surfactant with a tetradecyl tail, the lowest PdI values were observed for both homologous series. A PdI value below 0.3 is enough for phospholipid vesicles [46]. But in the region of the PdI from 0 to 0.3, there is a narrow interval of PdI values below 0.2, in which highly monodisperse particles are present [47]. Therefore, the tetradecyl homologue was selected after comprehensive evaluation of the zeta potential and PdI values.

**Figure 2:**
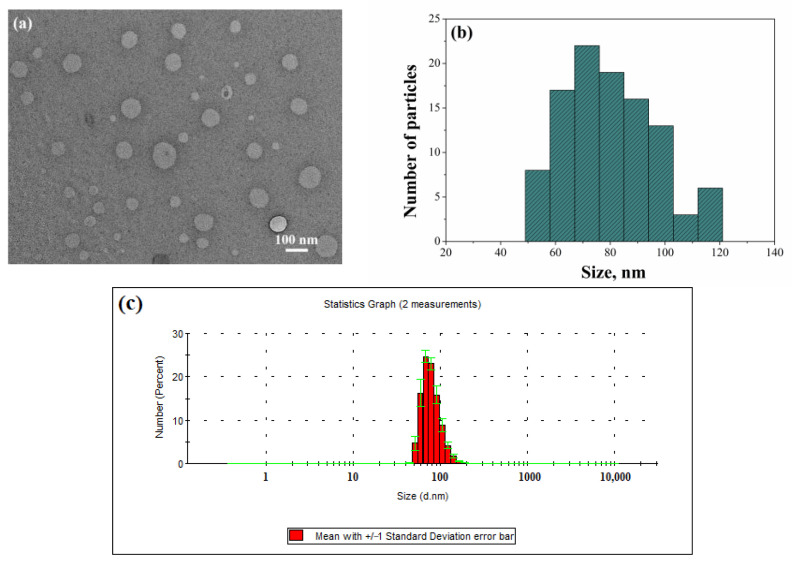
(**a**) Micrograph of liposomes obtained by TEM; (**b**) size distribution of liposomes in a TEM micrograph by number obtained using ImageJ software; (**c**) number-averaged size distribution of particles determined by DLS for PC/Chol/TPPB-14 at a molar ratio of 50/1, 25 °C.

To confirm the morphology and size of the obtained aggregates, microphotographs of the PC/Chol/TPPB-14 (50/1) liposomes were obtained using transmission electron microscopy (TEM). The microphotographs revealed the formation of spherical vesicles, mainly with a diameter of 80 ± 17 nm (Figure 2a,b). As can be seen, a slight polydispersity was observed in the system, which is also reflected in the diagram obtained by processing the photographs with the ImageJ software. Nevertheless, this is insignificant, and the data are in good agreement with the light scattering data (Figure 2c).

**Figure 3:**
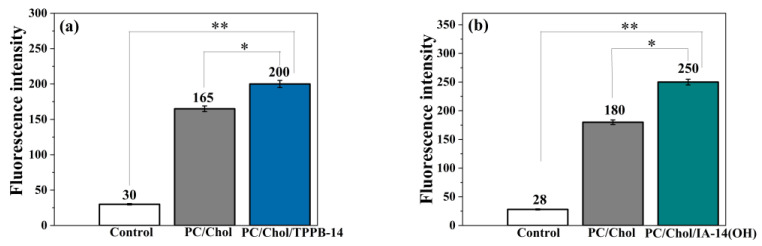
Cellular uptake of unmodified and modified (50/1) liposomes, i.e., (**a**) PC/Chol/TPPB-14 and (**b**) PC/Chol/IA-14(OH) by PANC-1 cells. Statistical analysis was performed using the Mann–Whitney test. (*) *p* < 0.01 compared to control; (**) *p* < 0.01 compared to PC Chol.

**Figure 4:**
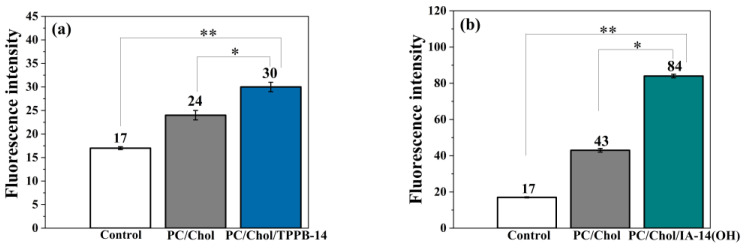
Cellular uptake of unmodified and modified (50/1) liposomes, i.e., (**a**) PC/Chol/TPPB-14 and (**b**) PC/Chol/IA-14(OH) by HuTu 80 cells. Statistical analysis was performed using the Mann–Whitney test. (*) *p* < 0.01 compared to control; (**) *p* < 0.01 compared to PC Chol.

The first step of assessing the biological activity of mitochondria-targeted nanoparticles is cellular internalization. A cellular uptake experiment was carried out by flow cytometry [48] using both unmodified and modified liposomes containing the fluorescent lipid 1,2-dioleoyl-sn-glycero-3-phosphoethanolamine-N-(lissamine rhodamine B sulfonyl) (ammonium salt) (DOPE-RhB) on the PANC-1 and HuTu 80 cell lines (Figure 3 and Figure 4). It can be observed that the fluorescence intensity within the cells demonstrated a discernible increase upon exposure to the liposomes, indicating a significant uptake of the liposomes by the PANC-1 and HuTu 80 cells compared with the control. However, in the case of the PC/Chol/TPPB-14 and PC/Chol/IA-14(OH) liposomes, this effect was more significant, indicating an enhanced penetrating ability of the modified liposomes. This can be explained in terms of liposome surface charge: positively charged liposomes interact with negatively charged cell membranes through electrostatic interactions, facilitating their internalization into cells [49]. A similar result was demonstrated by other researchers, which confirms the positive effect of nanoparticle cationization [50,51,52,53]. It is worth noting that the fluorescence intensity in the case of the HuTu 80 cell line was lower for all the systems compared to the PANC-1 cell line (Figure 3 and Figure 4). It can be explained in terms of the concentrations of the liposomes used for the experiment. The cytotoxicity of the investigated systems was higher in the case of the HuTu 80 cell line compared to the PANC-1 cells. For experiments involving the determination of the cellular uptake of liposomes, it is necessary to dilute the liposomes to concentrations below the IC_50_ values to ensure cell viability. In the case of the HuTu 80 cell line, the liposomes were diluted much more compared to the PANC-1 cell line, thereby leading to a reduction in the amount of fluorescent probe in the system, which in turn results in a reduced fluorescence intensity of the liposomes inside the cells.

A qualitative analysis of the cellular uptake was also conducted using fluorescence microscopy for the PC/Chol and PC/Chol/TPPB-14 (50/1) systems as an example (Figure S2). The blue fluorescence in the presented images corresponds to the localization of DAPI, which can bind strongly to adenine and thymine-rich areas of DNA, thereby identifying the cell nuclei. The red fluorescence reflects the localization of the liposomes with the fluorescent lipid DOPE-RhB. According to the obtained results, the PC/Chol and PC/Chol/TPPB-14 liposomes did indeed have the ability to penetrate the cells and localize around the nuclei. However, this effect was more pronounced in the case of the modified liposomes, as also demonstrated in Figure 3 and Figure 4.

**Figure 5:**
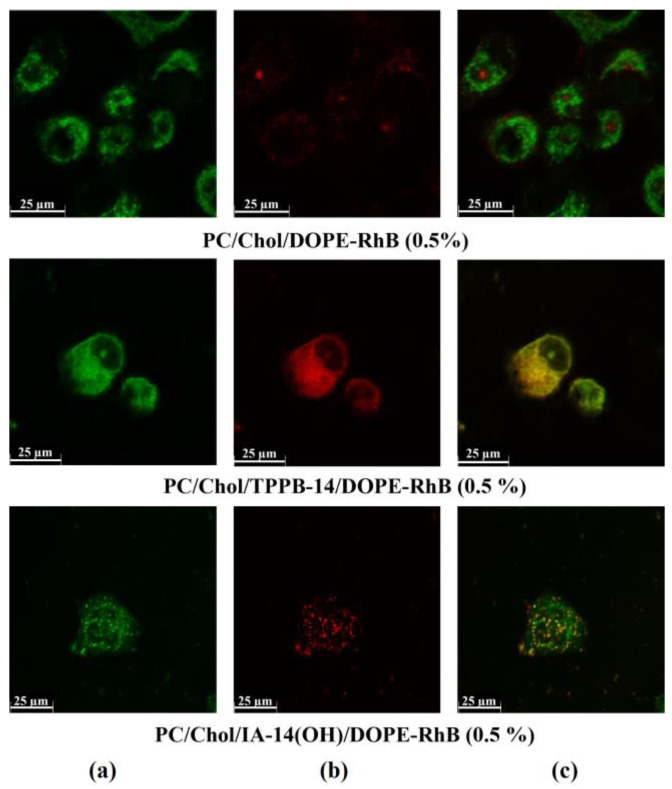
Colocalization analysis of PC/Chol/DOPE-RhB, PC/Chol/TPPB-14/DOPE-RhB, and PC/Chol/IA-14(OH)/DOPE-RhB liposomes (molar ratio is 50/1) with mitochondria of PANC-1 cells: (**a**) Mito-Tracker Green FM dye fluorescence (mitochondria); (**b**) DOPE-RhB fluorescence (liposomes); (**c**) the merged yellow image indicates colocalization of two probes. The concentration of DOPE-RhB is equal to 0.5% of the concentration of PC/Chol.

**Figure 6:**
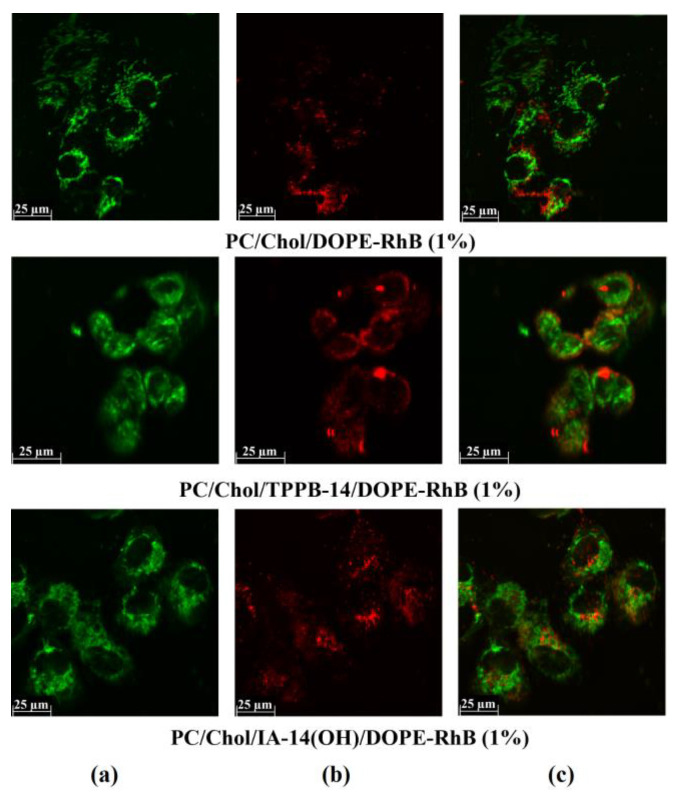
Colocalization analysis of PC/Chol/DOPE-RhB, PC/Chol/TPPB-14/DOPE-RhB, and PC/Chol/IA-14(OH)/DOPE-RhB liposomes (molar ratio is 50/1) with mitochondria of HuTu 80 cells: (**a**) Mito-Tracker Green FM dye fluorescence (mitochondria); (**b**) DOPE-RhB fluorescence (liposomes); (**c**) the merged yellow image indicates colocalization of two probes. The concentration of DOPE-RhB is equal to 1% of the concentration of PC/Chol.

The modification of liposomes with cationic amphiphile with delocalized positive charge enhances the ability of the resulting systems to penetrate the internal membrane of organelles [14,54]. To determine the potential use of PC/Chol/TPPB-14 and PC/Chol/IA-14(OH) liposomes for drug delivery, their ability to colocalize with mitochondria was investigated (Figure 5 and Figure 6). The HuTu 80 and PANC-1 cell lines were selected for the experiment. The mitochondria of the living cells were stained with Mito-Tracker Green FM (Figure 5a and Figure 6a), the colocalization of which with DOPE-RhB (Figure 5b and Figure 6b) indicates that the liposomes reached the mitochondria of the tumor cells. A qualitative analysis of the fluorescence signal distribution in the images obtained through confocal microscopy revealed a greater uptake of the modified liposomes by the cell mitochondria compared with the unmodified liposomes, which can be seen from the superimposition of the red and green channels, giving a yellow color (Figure 5c and Figure 6c).

**Figure 7:**
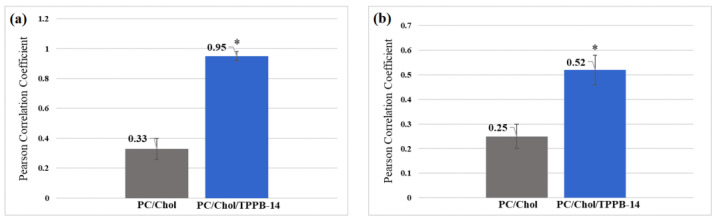
PCC for PC/Chol and PC/Chol/TPPB-14 liposomes on the (**a**) PANC-1 and (**b**) HuTu 80 cell lines. *—statistically significant result.

**Figure 8:**
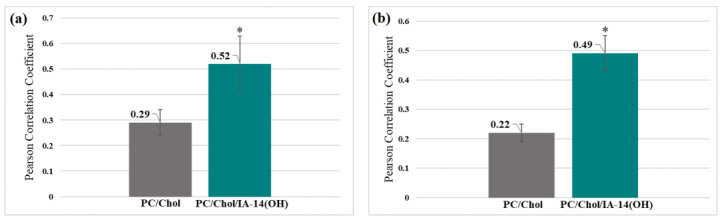
PCC for PC/Chol and PC/Chol/IA-14(OH) liposomes on the (**a**) PANC-1 and (**b**) HuTu 80 cell lines. *—statistically significant result.

To quantitatively assess the degree of colocalization, the Pearson Correlation Coefficient (PCC) was calculated. The PCC, a measure of the linear relationship between variables, ranges from −1 to 1. In this scale, −1 signifies a negative linear correlation, 0 denotes no correlation, and 1 indicates a positive correlation [55]. As evident from the presented diagrams, the PCC values for the PC/Chol/TPPB-14 and PC/Chol/IA-14(OH) liposomes were higher compared with the values for the unmodified liposomes for both the HuTu 80 and PANC-1 cell lines (Figure 7 and Figure 8). It is worth noting that the difference in colocalization efficiency between the liposomes modified with TPPB-14 and IA-14(OH) was minimal, except for the PANC-1 cell line: the colocalization degree of the PC/Chol/TPPB-14 liposomes was significantly higher than that of the PC/Chol/IA-14(OH) liposomes (PCC = 0.95 ± 0.03 and 0.52 ± 0.11, respectively) (Figure 7a and Figure 8a).

**Figure 9:**
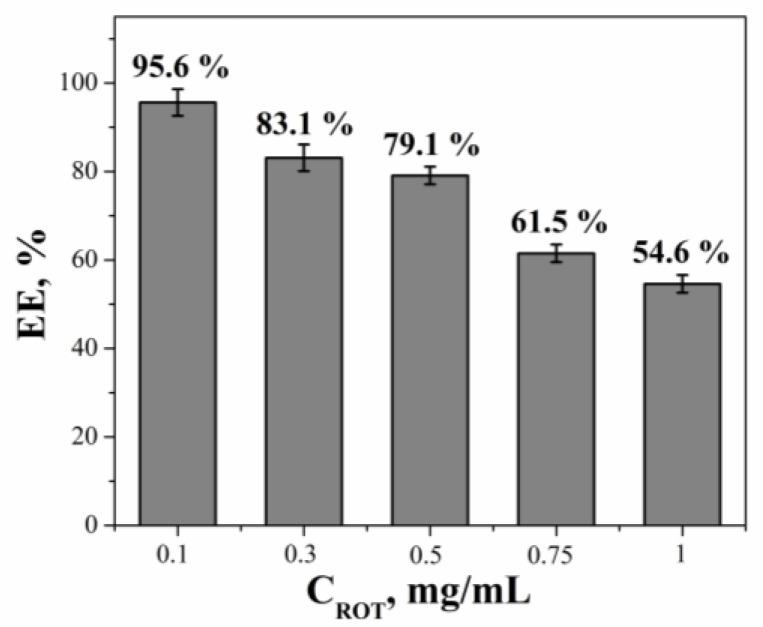
Encapsulation efficiency values of ROT in PC/Chol/TPPB-14 (50/1) liposomes, 25 °C.

One of the approaches in the treatment of chemotherapy-resistant oncological diseases involves the use of oxidative phosphorylation blockers, also known as mitochondrial poisons [39]. The combination of such mitochondrial-targeted nanoscale drug delivery systems with mitochondrial poisons represents a promising direction [56,57]. One such compound is rotenone (ROT), an isoflavonoid commonly used as a pesticide for animals, agricultural crops, and fisheries management [58]. It is known that ROT has the ability to inhibit the mitochondrial function of cells by inhibiting the mitochondrial complex Ⅰ, which is widely used to model Parkinson’s disease in laboratory animals [59,60,61], as well as to study the role of the mitochondrial respiratory chain during apoptosis [62]. Some researchers have demonstrated the ROT effectiveness in triggering apoptosis of tumor cells through the production of reactive oxygen species in cells [63,64]. Despite these properties, ROT has not found widespread use as a conventional drug for the treatment of cancer. This might be attributed to its high activity, non-selective action, and toxicity toward normal cells. In this regard, its encapsulation in liposomal systems targeting the mitochondria of tumor cells can significantly reduce its toxicity to normal cells. Therefore, at the next stage, ROT was loaded into the cationic liposomes, with a primary focus on determining the optimal loading concentration and physicochemical characteristics of liposomes. For this purpose, the extinction coefficient of ROT in various media was first determined (Table S1). Figure 9 demonstrates the encapsulation efficiency (EE) values for five different concentrations of ROT in the PC/Chol/TPPB-14 (50/1) liposomes as an example. According to the results, the most optimal loading concentration of ROT was 0.1 mg/mL. It is worth noting that at concentrations above 0.1 mg/mL, liposomes can encapsulate more ROT. However, this leads to inefficient use of the drug substance, which is economically impractical, and may affect the stability of liposomes and toxicity toward normal cells. Therefore, maintaining the optimal concentration of 0.1 mg/mL was a reasonable choice for further studies.

Because the incorporation of a hydrophobic substrate into liposomes can directly impact the physicochemical properties of the nanocarriers, the physicochemical characteristics of ROT-loaded nanocarriers were monitored over time (Table 2). At this stage of the study, attention was focused on two lipid/surfactant molar ratios, namely, 50/1 and 35/1. This is due to the fact that an increase in the surfactant concentration can adversely affect the toxicity of the entire formulation (Table S2) [65]. There was no noticeable effect of ROT on the physicochemical characteristics of the liposomes; the particle diameter was approximately 110 nm. The zeta potential of both the empty and ROT-loaded liposomes also converged almost completely (Table 2). As in the case of the empty liposomes, the zeta potential of the TPPB-n of the liposomes increased after 2 months of storage, while the zeta potential of the IA-n(OH) liposomes, on the contrary, decreased (Table 2), which confirms the pattern described earlier (Table 1). Similar results were obtained for the PC/Chol/IA-n(OH) system at a molar ratio of the components of 25/1 (Table S3). Although liposomes loaded with hydrophobic substrates tend to be less stable [66], the liposomes with ROT showed good stability over time, with the PdI for many systems below 0.1. In addition to the DLS data, the efficiency of the liposome encapsulation toward the ROT was determined for all the systems (Table 2). It has been shown that the surfactant hydrocarbon tail length and the lipid/surfactant ratio had an insignificant effect on the EE, and the values exceeded 90%.

**Figure 10:**
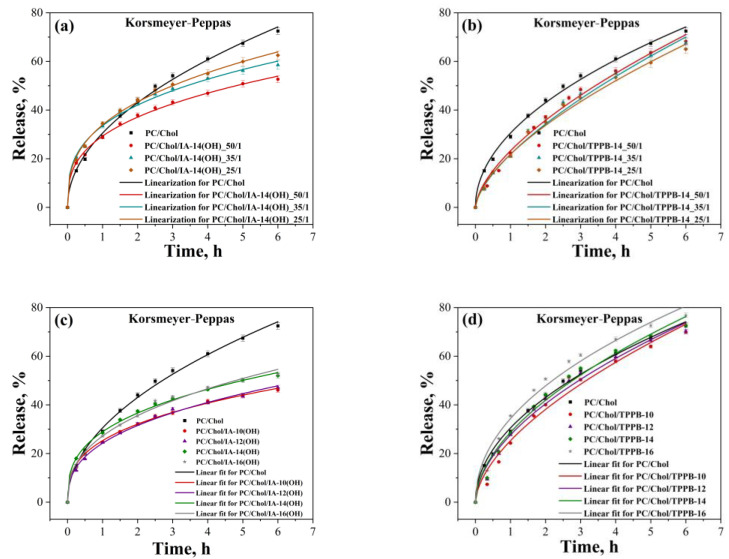
The Korsmeyer–Peppas kinetic model fitting curves of ROT release from unmodified and modified liposomes by varying (**a**) the molar ratio of PC/Chol/IA-14(OH); (**b**) the molar ratio PC/Chol/TPPB-14; (**c**) the IA-n(OH) hydrocarbon tail length at molar ratio of 50/1; (**d**) the TPPB-n hydrocarbon tail length at molar ratio of 50/1, 37 °C.

In the next stage, the ROT release rate from the modified liposomes was investigated using the dialysis method [67,68,69] (Figure 10). It is worth noting that studying the release of hydrophobic substrates is a challenging task because they are insoluble in water, and it is not possible to recreate biological conditions in vitro. Therefore, a sodium phosphate buffer (PBS) (pH = 7.4) and ethanol in a 1:1 ratio were chosen as the release medium. To ensure that the liposomes remained stable in such an environment, the size and PdI of the liposomes in water and in the PBS:ethanol medium were first determined using PC/Chol/ROT and PC/Chol/TPPB-14/ROT systems as the examples (15 mM, 50/1) (Figure S3). As seen from the graph, the size of the liposomes in the aqueous solution was in the range of 100 nm, with a polydispersity index of 0.225 ± 0.005 for PC/Chol/ROT and 0.142 ± 0.002 for PC/Chol/TPPB-14/ROT. Upon addition of the liposomes into the PBS:ethanol medium, the liposome size increased two-fold, but the systems remained monodispersed with PdI values below 0.3 (0.230 ± 0.007 for PC/Chol/ROT and 0.226 ± 0.003 for PC/Chol/TPPB-14/ROT) (Figure S3). It is worth noting that the liposome characteristics in the PBS:ethanol were easily detected, and there were no signs of aggregate destruction in the light scattering correlation plots. As seen in Figure 10, the ROT was released more rapidly from the unmodified liposomes compared with the modified systems. This effect was more pronounced in the case of the liposomes modified with the imidazolium surfactants (Figure 10a,c). The release rate of ROT from the IA-14(OH) liposomes depends on the surfactant concentration: a higher concentration led to an increased release rate (Figure 10a). Presumably, in this case, the higher surfactant concentration results in greater destabilization of the lipid bilayer and subsequent ROT leakage from the lipid bilayer. A similar assumption was made by other researchers in the context of liposome stability in the presence of hydrophilic fluorescein and hydrophobic rhodamine [66]. Interestingly, in the case of TPPB-14 and IA-10(OH), there was no statistically significant impact of the surfactant concentration on the ROT release rate (Figure 10b and Figure S4, respectively). Meanwhile, more noticeable differences were observed when varying the surfactant hydrocarbon tail length. For both IA-n(OH) and TPPB-n, increasing the length of the surfactant alkyl tail led to an increase in the ROT release rate (Figure 10c,d). This phenomenon can also be explained in terms of a decrease in the critical micelle concentration with an increase in the surfactant hydrocarbon tail length [70,71], which can lead to an increase in the ability of the surfactants to loosen the lipid bilayer and leakage of ROT from liposomes [72,73,74]. However, it should be noted that the differences between the surfactants with C_16_ and C_10_ did not exceed 10%. Considering the fact that the release profiles of ROT change and depend on the liposome composition, it can be concluded that the liposomes remain stable during dialysis, and ethanol does not significantly contribute to the rate of substrate release. Otherwise, the ROT release profiles from the dialysis bag would be identical because free ROT would release from the dialysis bag after liposome disruption.

For a more detailed understanding of the kinetics of the ROT release from the liposomes, the obtained dependencies were processed by two kinetic models, namely, the Korsmeyer–Peppas and Higuchi models (Figure 10 and Figure S5, respectively). Based on personal experience and the literature data, these two models have proven most suitable for characterizing the kinetics of substrate release from liposomes [36,73,75,76,77,78]. According to the presented graphs (Figure 10), the Korsmeyer–Peppas model better described the release profiles of ROT from the modified liposomes than the Higuchi model (Figure S5), because the correlation coefficient (R^2^) in all the cases exceeded 0.98 (Table 3). For the IA-10(OH) liposomes, this trend was also confirmed (Table S4). The presented values of the rate constant confirm the judgments made above: an increase in the concentration of IA-14(OH) led to a slight increase in the ROT release rate, as well as an increase in the length of the surfactant hydrocarbon tail. Interestingly, based on the diffusion release exponent values (n), the mechanisms of the ROT release from the PC/Chol/IA-n(OH) and PC/Chol/TPPB-n liposomes were different. The PC/Chol/IA-n(OH) liposomes were characterized by Fickian diffusion (n ˂ 0.45), while for the PC/Chol/TPPB-n liposomes, the release mechanism changed to non-Fickian diffusion (0.45 ˂ n ˂ 0.89) [79,80], which means that in the case of liposomes modified with triphenylphosphonium surfactants, a synergistic effect of diffusion of the drug substance and the dissolution of the phospholipid bilayer of liposomes is observed during the release [81]. The absorption spectra of ROT for all the systems are shown in Figures S6–S11.

The main challenge in the field of cancer treatment is the insufficient selectivity of the systems toward tumor cells [82,83]. Therefore, the next step of the biological activity study was focused on evaluating the cytotoxicity of the ROT-loaded liposomes toward the tumor and normal cell lines in vitro. For this purpose, the HuTu 80, PANC-1, Chang liver, and WI-38 cell lines were selected. As evident from the data presented in Table 4, free ROT exhibited a considerably high cytotoxicity toward the tumor cells (the IC_50_ was 2.8 μM). For comparison, IC_50_ values for doxorubicin are also within the micromolar range depending on the cell line [84]. It is important to note that free ROT exhibited a sufficiently high selectivity index (SI > 173) toward the HuTu 80 cell line, which increased upon encapsulation into the PC/Chol/TPPB-14 liposomes (SI = 307). This result indicates a synergistic effect of the combination of the modified TPPB-14 liposomes with ROT. It is worth noting that the cytotoxicity of TPPB-n was also quite high, with the maximum selectivity index observed for TPPB-12 and TPPB-14 (Table S2). For all the modified systems, a sufficiently high selectivity toward the tumor cells was observed. However, the SI values for the modified liposomes did not exceed the SI of free ROT (Table 4). Additionally, a significant finding is that the IC_50_ values of the investigated formulations toward the HuTu 80 cell line were much lower than those for the PANC-1 cell line. This suggests that the effectiveness of nanoparticles depends not only on their physicochemical characteristics but also on the cell type [85].

**Figure 11:**
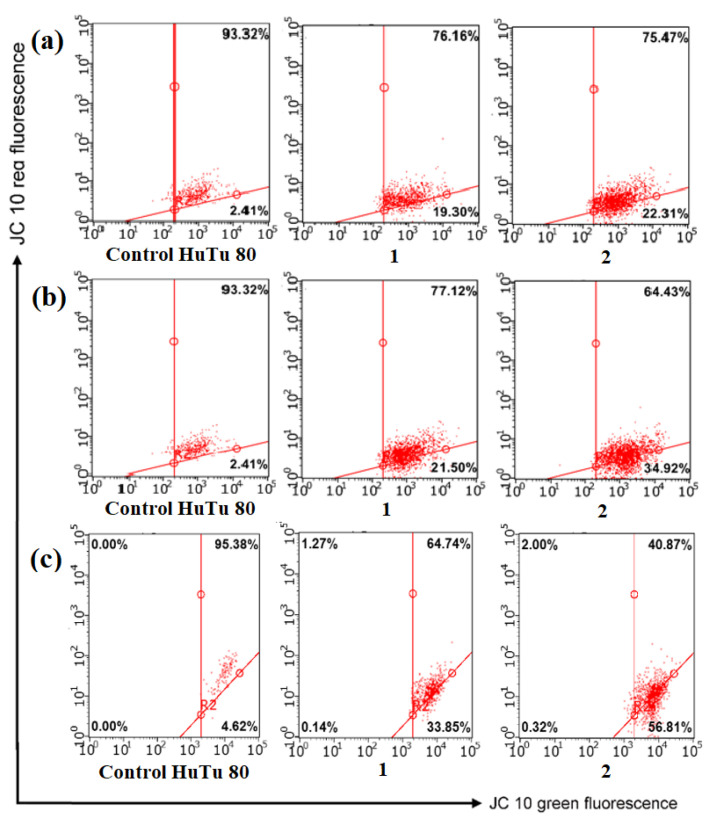
Analysis of apoptosis induction of HuTu 80 tumor cell line treated with (**a**) PC/Chol/ROT; (**b**) PC/Chol/TPPB-14/ROT; (**c**) PC/Chol/IA-14(OH)/ROT. (1) IC_50_/2; (2) IC_50_.

Following the confirmation of the mitochondria-targeting activity of the PC/Chol/TPPB-14 and PC/Chol/IA-14(OH) liposomes, along with the high selective cytotoxic activity of the ROT-loaded liposomes, the investigated formulations were tested to validate their apoptotic activity. To assess the liposome ability to induce apoptosis via the mitochondrial pathway, the pro-apoptotic properties of the investigated systems were evaluated using flow cytometry on the HuTu 80 cell line, using the fluorescent dye JC-10 (Figure 11). JC-10 accumulates in the mitochondrial matrix and forms aggregates (J-aggregates) with red fluorescence in normal cells with high mitochondrial membrane potential. The membrane potential decreases in apoptotic cells, and JC-10 starts to diffuse out of the mitochondria, converting into a monomeric form (J-monomer) and emitting green fluorescence [86,87]. According to the results, a dose-dependent reduction in the mitochondrial membrane potential was observed after treating the HuTu 80 cells with the liposomal formulations. The process of apoptosis induction became more pronounced with the addition of the PC/Chol/TPPB-14/ROT and PC/Chol/IA-14(OH)/ROT liposomes. The obtained results suggest that the cytotoxic mechanism of the tested systems is attributed to the induction of apoptosis via the intrinsic mitochondrial pathway.

Thus, it should be noted that the work once again confirmed the fact that not only traditional triphenylphosphonium conjugates have mitotropic activity but also other surfactants with a delocalized positive charge, namely, surfactants with an imidazolium head group, possess such functionality [34]. The significance of this article is testing the approach of replacing traditional chemotherapeutic drugs with mitochondrial poisons in the treatment of resistant forms of cancer, proposed by Viale et al. [39], using modified liposomes. In this context, rotenone can be considered as a commercially available model substrate to work out all the stages of the experiment. It is worth noting that rotenone loaded into liposomal nanocontainers was studied both from a physicochemical point of view (the effect on liposome encapsulation efficiency, the release rate, and the stability of liposomes) and from a biological point of view (determining the IC_50_ values and the apoptosis pathway). The obtained results provide a full basis for continuing experiments with the formulations studied in the direction of in vivo research.

## 3. Materials and Methods

### 3.1. Chemicals

L-α-phosphatidylcholine (95%) and fluorescent lipid 1,2-dioleoyl-sn-glycero-3-phosphoethanolamine-N-(lissamine rhodamine B sulfonyl) (ammonium salt) (DOPE-RhB, >99%) were purchased from Avanti Polar Lipids, Inc. (Alabaster, AL, USA). Cholesterol (≥99%) was purchased from Sigma-Aldrich (St. Louis, MO, USA). Homologous series of IA-n(OH) (n = 10, 12, 14, 16) and TPPB-n (n = 10, 12, 14) amphiphiles were synthesized according to the published methods [70,71]. Hexadecyltriphenylphosphonium bromide (TPPB-16, ≥98%) was purchased from Alfa Aesar (Haverhill, MA, USA). Rotenone (>95%) was purchased from Tokyo Chemical Industry Co., Ltd. (Tokyo, Japan). MitoTracker Green FM (98%) was used to stain mitochondria of living cells (Thermo Fisher Scientific, Waltham, MA, USA). For cellular uptake assay, 4′,6-diamidino-2-phenylindole (DAPI) was used (Sigma-Aldrich, St. Louis, MO, USA). Sodium phosphate buffer (PBS) was purchased from UralChemInvest (Ufa, Russia). Chloroform and ethanol (HPLC) were purchased from JSC №1 BASE Chemical reagents (Staraya Kupavna, Russia). Liposomal dispersions were prepared using ultrapure Milli-Q water purified by Simplicity^®^ UV system (Millipore SAS, Molsheim, France).

### 3.2. Liposome Preparation

Liposomes were obtained by lipid film hydration method according to the algorithm published in [88] at a PC/Chol molar ratio of 9/1 (total concentration was 15 mM). Liposomes were modified by incorporating TPPB-n and IA-n(OH) with 10, 12, 14, and 16 carbon atoms in hydrocarbon tail into the lipid bilayer at surfactant/lipid ratio of 50/1, 35/1, and 25/1. ROT was also incorporated into the lipid bilayer at the stage of lipid film formation. The lipid film was then hydrated with Milli-Q water, incubated in a water bath at 60 °C within 1 h, followed by 5 cycles of freezing and thawing using liquid nitrogen. The resulting dispersions were then extruded through a polycarbonate membrane using an LiposoFast Basic extruder (Avestin, Ottawa, ON, Canada) to obtain aggregates with diameter of 100 nm. Liposomes were stored at 4 °C.

### 3.3. Dynamic and Electrophoretic Light Scattering (DLS/ELS)

Physicochemical characteristics of liposomes diluted to 2 mM, i.e., hydrodynamic diameter (D_h_), polydispersity index (PdI), and zeta potential (ζ), were determined on Malvern ZetaSizer Nano instrument (Malvern Instruments Ltd., Worcestershire, UK). The hydrodynamic diameter and zeta potential were calculated automatically using the Stokes–Einstein and Smoluchowski equations, respectively, as presented in [43].

### 3.4. Transmission Electron Microscopy (TEM)

Liposomes with concentration of 5 μM were visualized using transmission electron microscopy on a Hitachi HT 7700 Exalens instrument (Hitachi, Tokyo, Japan) at an accelerating voltage of 100 kV. Sample was deposited on copper grid (Ted Pella, Pella, IA, USA) with a carbon-formvar support film and dried at room temperature within 2 h. Diameter of liposomes was then analyzed using ImageJ software (version number 1.53t).

### 3.5. Encapsulation Efficiency (EE) and Release Rate of Substrate

The extraction method of unencapsulated substrate in ethanol was used to evaluate the liposome encapsulation efficiency toward ROT [89,90]. The ROT concentration was determined spectrophotometrically on Specord 250 Plus (Analytik Jena AG, Jena, Germany) using a 0.5 cm quartz cuvette (Hellma Analytics, Müllheim, Germany). Encapsulation efficiency was calculated using the following equation:

$$\mathrm{EE}=\frac{\mathrm{Total}\;\mathrm{amount}\;\mathrm{of}\;\mathrm{substrate}-\mathrm{Amount}\;\mathrm{of}\;\mathrm{free}\;\mathrm{substrate}}{\mathrm{Total}\;\mathrm{amount}\;\mathrm{of}\;\mathrm{substrate}}\times 100$$

The ROT release rate from modified liposomes was analyzed using the dialysis method. For this purpose, liposomal dispersion was placed in a dialysis bag with a pore size of 3.5 kDa. Dialysis was carried out in PBS:ethanol medium (1:1) at 37 °C, with a stirring speed of 250 rpm. Aliquots (2 mL) were taken from the dialysis medium at fixed time intervals and absorption spectra were recorded using Specord 250 Plus (Analytik Jena AG, Jena, Germany). After measurement the aliquot was returned to the dialysis medium. The absorption maximum of ROT was detected at λ = 295 nm. The extinction coefficient of ROT in PBS:ethanol medium was equal to 16606 M^−1^⋅cm^−1^ (Table S1). The graphs represent the percentage of substrate release as the average of at least three experiments, with a standard deviation of less than 3% for all systems. Release profiles were fitted to Korsmeyer–Peppas and Higuchi models using OriginPro 8.5 software (OriginLab Corporation, Northampton, MA, USA) according to the equations presented in [36].

### 3.6. Cell Culture

HuTu 80 (duodenal adenocarcinoma), PANC-1 (pancreatic carcinoma), and WI-38 (normal embryonic lung cells) cell lines were obtained from the collection of type cultures of the Institute of Cytology (The Russian Academy of Sciences, Saint Petersburg, Russia). Chang liver (normal liver cells) cell line was purchased from D.I. Ivanovskiy Institute of Virology (N.F. Gamaleya National Research Center of Epidemiology and Microbiology of the Ministry of Health, Moscow, Russia). Cells were seeded on a 96-well Nunc plate with a density of 5 × 10^3^ cells per well using 100 μL of standard Eagle’s medium. The plate was then placed in a CO_2_ incubator at 37 °C until a monolayer of cells was formed.

### 3.7. Cellular Uptake

Cellular uptake of liposomes was analyzed using flow cytometry. Cells were seeded into 24-well plates (Eppendorf, Hamburg, Germany) at a concentration of 1 × 10^5^ cells per well. After 24 h of incubation, liposomes with DOPE-RhB were added to the wells. Cells were then incubated for 24 h in a CO_2_ incubator. Cellular uptake of liposomes was analyzed using Guava EasyCyte 8HT flow cytometer (Merck KGaA, Darmstadt, Germany). After that, cells were fixed, and nuclei were stained with DAPI. The survey was carried out using a Nikon Eclipse Ci-S fluorescence microscope (Nikon, Tokyo, Japan). Untreated cells were used as a negative control. A more detailed description of the experiment is published in [34].

### 3.8. Cytotoxicity

The cytotoxicity of liposomes toward cancer and normal cells was determined using the colorimetric MTT test. Liposomes at various dilutions were added directly to the wells with cells after removing the nutrient medium for 24 h incubation. After that, the culture medium was removed from wells with following addition of 100 µL of serum-free nutrient medium containing (3-(4,5-dimethylthiazol-2-yl)-2,5-diphenyl-tetrazolium bromide) (NeoFroxx GmbH, Einhausen, Germany) at a concentration of 0.5 mg/mL. Cells were then incubated within 4 h at 37 °C. Optical density of medium was recorded at 540 nm on an InVitroLogic microplate reader (Medico-Biological Union, Novosibirsk, Russia). Calculation of the liposome concentration that causes growth inhibition of 50% of cells (IC_50_) was made using MLA—“Quest Graph™ IC_50_ Calculator” (AAT Bioquest, Inc., Pleasanton, CA, USA) [91]. A more detailed description of the experiment is published in [92]. Experiments for all compounds were carried out in triplicate.

### 3.9. Colocalization Assay

Cells were seeded in 35 mm × 35 mm glass plates. After a 24 h incubation, the nutrient medium was changed to liposomal dispersion with DOPE-RhB and cells were further incubated for 24 h. Then, cells were washed twice with PBS and incubated for 20 min in a medium containing Mito-Tracker Green FM to stain the mitochondria. The fluorescence of dyes was visualized using a Leica SP5 TCS confocal scanning microscope (Leica Microsystems, Wetzlar, Germany). The fluorescence emission of DOPE-RhB and MitoTracker Green FM was collected at 570–700 nm and at 500–540 nm, respectively. The Pearson Correlation Coefficient (PCC) was used to quantify the correlation between the fluorescence intensities of two dyes, i.e., colocalization.

### 3.10. Mitochondrial Membrane Potential

Cells were seeded in 6-well plates at 1 × 10^6^ cells per well and incubated for 24 h with liposomes. Cells were harvested at 2000 rpm for 5 min, washed twice with cold PBS, then resuspended in JC-10 (10 µg/mL) (Sigma-Aldrich, St. Louis, MO, USA). After a 10 min incubation at 37 °C, cells were washed three times and suspended in PBS. Apoptosis induction was studied using Guava EasyCyte 8HT flow cytometer (Merck KGaA, Darmstadt, Germany).

### 3.11. Statistics

All data processing was performed using Microsoft Excel 2016^®^ (Microsoft, Redmond, WA, USA) and OriginPro 8.5 (OriginLab Corporation, Northampton, MA, USA). Results are expressed as the mean ± standard deviation. Analysis of the diameter of the particles obtained by TEM was performed using ImageJ software (version number 1.53t). Statistical analysis of cellular uptake results was performed using the Mann–Whitney test. Significance was tested at the 0.05 level of probability (*p*).

## 4. Conclusions

New mitochondria-targeted liposomes based on soy phosphatidylcholine, cholesterol, and triphenylphosphonium/imidazolium amphiphiles with various hydrocarbon tail lengths (10, 12, 14, and 16) and lipid/surfactant molar ratios (50/1, 35/1, and 25/1) have been developed for the treatment of oncological diseases resistant to traditional types of chemotherapy. Their physicochemical characteristics were investigated, and their stability was demonstrated for more than 2 months, even in systems with a low concentration of cationic amphiphiles. Liposomes were characterized by a size of about 100 nm with high monodispersity (the PdI did not exceed 0.25) and high zeta potential (>+ 30 mV). The optimal concentration of mitochondrial poison ROT was also selected (0.1 mg/mL) for loading into liposomes, at which the efficiency of ROT encapsulation exceeded 90%. The ROT-loaded liposomes noncovalently modified with cationic surfactants showed high cytotoxicity toward pancreatic carcinoma (PANC-1) and duodenal adenocarcinoma (HuTu 80) cells with the selectivity index (SI) of 307 compared with the normal Chang liver cell line. It has been shown by confocal microscopy and flow cytometry that the modification of liposomes with triphenylphosphonium and imidazolium lipophilic cation provides a higher degree of penetration and colocalization with tumor cell mitochondria compared with unmodified carriers, inducing cell apoptosis via the internal mitochondrial pathway. Thus, these results suggest that the combination of new mitochondria-targeted liposomes with the mitochondrial poison ROT is a promising strategy for oncological disease treatment, which can be further tested under in vivo conditions.

## Figures and Tables

**Table 1:** Physicochemical properties of empty PC/Chol/TPPB-n and PC/Chol/IA-n(OH) liposomes at various lipid/surfactant molar ratio, 4 °C.

Formulation	D_h,_ nm	PdI	ζ, mV	D_h,_ nm	PdI	ζ, mV
	1st Day			2 Months		
PC	131 ± 1	0.214 ± 0.013	+1.8 ± 0.9	115 ± 1	0.105 ± 0.008	−13 ± 1
PC/Chol	133 ± 5	0.215 ± 0.022	−7.0 ± 0.2	112 ± 1	0.118 ± 0.023	−14 ± 1
50/1						
PC/Chol/TPPB-10	152 ± 1	0.239 ± 0.003	+29 ± 2	114 ± 2	0.102 ± 0.010	+31 ± 3
PC/Chol/TPPB-12	102 ± 1	0.116 ± 0.018	+30 ± 2	100 ± 1	0.145 ± 0.008	+35 ± 2
PC/Chol/TPPB-14	120 ± 1	0.085 ± 0.012	+33 ± 2	106 ± 1	0.086 ± 0.012	+37.2 ± 0.5
PC/Chol/TPPB-16	98 ± 1	0.103 ± 0.018	+38 ± 2	120 ± 1	0.124 ± 0.010	+44 ± 1
PC/Chol/IA-10(OH)	150 ± 1	0.200 ± 0.012	+10.5 ± 0.2	96 ± 2	0.078 ± 0.013	+3.3 ± 0.4
PC/Chol/IA-12(OH)	104 ± 1	0.177 ± 0.002	+26 ± 1	116 ± 4	0.269 ± 0.037	+13 ± 1
PC/Chol/IA-14(OH)	111 ± 1	0.083 ± 0.012	+39 ± 1	113 ± 1	0.090 ± 0.011	+27 ± 1
PC/Chol/IA-16(OH)	109 ± 1	0.105 ± 0.011	+44.2 ± 0.4	121 ± 1	0.168 ± 0.006	+25.1 ± 0.5
35/1						
PC/Chol/TPPB-10	110 ± 1	0.092 ± 0.023	+28.8 ± 0.6	122 ± 1	0.130 ± 0.021	+33 ± 1
PC/Chol/TPPB-12	104 ± 1	0.134 ± 0.014	+31 ± 1	103 ± 1	0.137 ± 0.022	+45 ± 3
PC/Chol/TPPB-14	109 ± 1	0.083 ± 0.012	+35 ± 1	108 ± 2	0.138 ± 0.019	+42 ± 1
PC/Chol/TPPB-16	102 ± 1	0.119 ± 0.008	+30.3 ± 0.3	122 ± 1	0.147 ± 0.012	+47 ± 4
PC/Chol/IA-10(OH)	106 ± 2	0.204 ± 0.034	+12.4 ± 0.6	99 ± 2	0.133 ± 0.028	+7.5 ± 0.5
PC/Chol/IA-12(OH)	94 ± 1	0.100 ± 0.015	+31 ± 1	113 ± 5	0.237 ± 0.015	+19 ± 2
PC/Chol/IA-14(OH)	110 ± 1	0.094 ± 0.002	+41 ± 1	116 ± 1	0.101 ± 0.014	+26 ± 3
PC/Chol/IA-16(OH)	107 ± 1	0.151 ± 0.004	+49.2 ± 0.4	132 ± 3	0.217 ± 0.004	+34 ± 3
25/1						
PC/Chol/TPPB-10	113 ± 1	0.076 ± 0.019	+39 ± 1	114 ± 1	0.101 ± 0.004	+38 ± 1
PC/Chol/TPPB-12	120 ± 1	0.137 ± 0.032	+42 ± 1	103 ± 1	0.114 ± 0.004	+43 ± 3
PC/Chol/TPPB-14	111 ± 1	0.133 ± 0.005	+38.8 ± 0.5	122 ± 1	0.124 ± 0.012	+47 ± 2
PC/Chol/TPPB-16	123 ± 1	0.097 ± 0.009	+40.3 ± 0.4	119 ± 2	0.103 ± 0.006	+53 ± 2
PC/Chol/IA-10(OH)	108 ± 1	0.203 ± 0.006	+15 ± 1	97 ± 2	0.120 ± 0.024	+6 ± 2
PC/Chol/IA-12(OH)	97 ± 1	0.132 ± 0.004	+35 ± 1	134 ± 3	0.255 ± 0.004	+25 ± 0.8
PC/Chol/IA-14(OH)	111 ± 1	0.101 ± 0.023	+45 ± 1	117 ± 1	0.103 ± 0.010	+35 ± 1
PC/Chol/IA-16(OH)	112 ± 1	0.094 ± 0.018	+51 ± 1	131 ± 4	0.207 ± 0.107	+38 ± 1

**Table 2:** Physicochemical characteristics of PC/Chol/TPPB-n and PC/Chol/IA-n(OH) liposomes loaded with ROT (0.1 mg/mL) at two lipid/surfactant molar ratios, 4 °C.

Formulation	EE, %	D_h_, nm	PdI	ζ, mV	D_h_, nm	PdI	ζ, mV
		1st Day			2 Months		
50/1							
PC/Chol/TPPB-10	91 ± 2	110 ± 1	0.103 ± 0.005	29 ± 1	121 ± 1	0.099 ± 0.017	29 ± 1
PC/Chol/TPPB-12	94 ± 1	119 ± 2	0.115 ± 0.011	31 ± 1	123 ± 2	0.115 ± 0.018	31 ± 1
PC/Chol/TPPB-14	94 ± 1	114 ± 1	0.109 ± 0.011	33 ± 1	115 ± 1	0.098 ± 0.027	31 ± 1
PC/Chol/TPPB-16	95 ± 2	116 ± 2	0.106 ± 0.005	25.5 ± 0.7	120 ± 2	0.098 ± 0.017	34 ± 3
PC/Chol/IA-10(OH)	97 ± 1	109 ± 1	0.068 ± 0.011	6.4 ± 0.5	125 ± 2	0.124 ± 0.007	3.2 ± 0.4
PC/Chol/IA-12(OH)	97 ± 1	109 ± 1	0.083 ± 0.004	25.4 ± 0.1	118 ± 1	0.146 ± 0.003	8.5 ± 0.1
PC/Chol/IA-14(OH)	96 ± 2	111 ± 1	0.075 ± 0.003	36.2 ± 0.5	124 ± 1	0.169 ± 0.012	14.3 ± 0.5
PC/Chol/IA-16(OH)	98 ± 1	110 ± 1	0.090 ± 0.003	39 ± 1	119 ± 1	0.160 ± 0.005	13.2 ± 0.5
35/1							
PC/Chol/TPPB-10	91 ± 3	114 ± 2	0.116 ± 0.015	31 ± 1	110 ± 2	0.096 ± 0.014	37.2 ± 0.3
PC/Chol/TPPB-12	92 ± 2	138 ± 3	0.250 ± 0.006	34 ± 2	106 ± 2	0.072 ± 0.008	44 ± 1
PC/Chol/TPPB-14	94 ± 1	122 ± 1	0.203 ± 0.019	35 ± 1	114 ± 1	0.082 ± 0.020	45 ± 3
PC/Chol/TPPB-16	93 ± 1	117 ± 1	0.172 ± 0.001	32 ± 1	120 ± 1	0.171 ± 0.013	48 ± 2
PC/Chol/IA-10(OH)	89 ± 2	109 ± 1	0.095 ± 0.007	9 ± 1	151 ± 3	0.282 ± 0.016	7.2 ± 0.2
PC/Chol/IA-12(OH)	94 ± 2	110 ± 2	0.109 ± 0.002	30.2 ± 0.2	117 ± 1	0.107 ± 0.025	24 ± 1
PC/Chol/IA-14(OH)	92 ± 1	118 ± 1	0.155 ± 0.013	40 ± 1	113 ± 2	0.127 ± 0.004	35 ± 1
PC/Chol/IA-16(OH)	94 ± 2	115 ± 2	0.133 ± 0.014	45.1 ± 0.2	115 ± 1	0.072 ± 0.008	28 ± 1

**Table 3:** The Korsmeyer–Peppas and Higuchi kinetic model fitting parameters of ROT release from PC/Chol/IA-n(OH) and PC/Chol/TPPB-n liposomes at various lipid/surfactant molar ratio.

Formulation	Lipid/Surfactant Molar Ratio	Korsmeyer–Peppas			Higuchi	
		k_KP_	n	R^2^	k_H_	R^2^
PC/Chol	-	30.52 ± 0.57	0.496 ± 0.014	0.9968	30.36 ± 0.24	0.9971
PC/Chol/IA-14(OH)	50/1	29.29 ± 0.35	0.340 ± 0.009	0.9973	24.26 ± 0.84	0.9288
	35/1	33.56 ± 0.47	0.325 ± 0.011	0.9961	27.31 ± 1.05	0.9098
	25/1	33.92 ± 0.44	0.354 ± 0.010	0.9971	28.51 ± 0.91	0.9415
PC/Chol/TPPB-14	50/1	23.24 ± 0.90	0.623 ± 0.028	0.9914	27.05 ± 0.73	0.9723
	35/1	23.18 ± 0.76	0.614 ± 0.023	0.9940	26.30 ± 0.82	0.9646
	25/1	23.15 ± 0.70	0.591 ± 0.022	0.9943	25.52 ± 0.70	0.9719
PC/Chol/IA-10(OH)	50/1	25.30 ± 0.35	0.344 ± 0.011	0.9964	21.03 ± 0.71	0.9317
PC/Chol/IA-12(OH)		24.23 ± 0.40	0.379 ± 0.013	0.9958	20.99 ± 0.56	0.9611
PC/Chol/IA-14(OH)		28.94 ± 0.35	0.341 ± 0.009	0.9973	23.96 ± 0.83	0.9288
PC/Chol/IA-16(OH)		27.18 ± 0.63	0.389 ± 0.018	0.9921	23.82 ± 0.62	0.9641
PC/Chol/TPPB-10		25.28 ± 1.50	0.593 ± 0.043	0.9782	28.32 ± 0.81	0.9682
PC/Chol/TPPB-12		27.66 ± 1.22	0.547 ± 0.032	0.9855	29.31 ± 0.58	0.9834
PC/Chol/TPPB-14		28.70 ± 1.27	0.546 ± 0.032	0.9853	30.34 ± 0.60	0.9834
PC/Chol/TPPB-16		34.38 ± 1.51	0.476 ± 0.033	0.9809	33.39 ± 0.65	0.9817

k_KP_ is the release constant taking into account the structural and geometric characteristics of the dosage form, %/min^n^; k_H_ is the Higuchi release constant, %/min^1/2^.

**Table 4:** Cytotoxicity and selectivity index of ROT in unmodified and modified liposomes (molar ratio is 50/1) toward normal and tumor cell lines.

Formulation	IC_50_, µM				SI_Chang Liver/HuTu 80_
	Tumor Cell Lines		Normal Cell Lines		
	HuTu 80	PANC-1	Chang Liver	WI-38	
ROT	2.8	>1000	484	1000	>173
PC/Chol	5.0	13.2	125	27.4	25
PC/Chol/TPPB-14	0.07	4.9	21.5	3.2	307
PC/Chol/IA-10(OH)	1.3	-	>63.5	-	>49
PC/Chol/IA-12(OH)	1	-	35	-	35
PC/Chol/IA-14(OH)	1	7.8	>63.5	-	>63.5
PC/Chol/IA-16(OH)	0.5	8.8	56.3	-	113

## Data Availability

The analyzed data are included in this manuscript. The raw data are available from the authors upon request.

## Supplementary Materials

The following supporting information can be downloaded at: https://www.mdpi.com/article/10.3390/molecules28207229/s1. Figure S1: Zeta potential of PC/Chol/TPPB-n and PC/Chol/IA-n(OH) liposomes by varying: (a) TPPB-n hydrocarbon tail length at a molar ratio of 50/1; (b) IA-n(OH) hydrocarbon tail length at a molar ratio of 50/1; (c) lipid/TPPB-14 molar ratio; (d) lipid/IA-14(OH) molar ratio on the preparation day, 25 °C.; Figure S2: Qualitative analysis of cellular uptake study of PC/Chol/DOPE-RhB and PC/Chol/TPPB-14/DOPE-RhB liposomes by HuTu 80 cells using fluorescence microscopy.; Table S1: Extinction coefficient values of ROT in various medium, 25 °C.; Table S2: Cytotoxicity and selectivity index of TPPB-n on normal and tumor cell lines.; Table S3: Physicochemical characteristics of PC/Chol/IA-n(OH) modified liposomes (25/1) loaded with ROT (0.1 mg/mL): encapsulation efficiency (EE), hydrodynamic diameter (Dh), polydispersity index (PdI) and zeta potential (ζ) over time, 4 °C.; Figure S3: Intensity averaged size distribution of PC/Chol/ROT and PC/Chol/TPPB-14/ROT liposomes in aqueous solution and in PBS:ethanol medium (1:1), 25 °C.; Figure S4: (a) Korsmeyer-Peppas and (b) Higuchi kinetic model fitting curves of ROT release from PC/Chol/IA-10(OH) liposomes at various molar ratio of components, 37 °C.; Figure S5: The Higuchi kinetic model fitting curves of ROT release from modified liposomes by varying: (a) the molar ratio of PC/Chol/IA-14(OH); (b) the molar ratio PC/Chol/TPPB-14; (c) the IA-n(OH) hydrocarbon tail length at molar ratio of 50/1; (d) the TPPB-n hydrocarbon tail length at molar ratio of 50/1, 37 °C.; Table S4: The Korsmeyer-Peppas and Higuchi kinetic model fitting parameters of ROT release from PC/Chol/IA-10(OH) liposomes at various lipid/surfactant molar ratio.; Figure S6: The absorption spectra of ROT at different time intervals of release for PC/Chol. PBS:ethanol (1:1), 37 °C; cuvette thickness = 1 cm; the arrow indicates the direction of dialysis duration increasing.; Figure S7: The absorption spectra of ROT at different time intervals of release for PC/Chol/IA14(OH) at molar ratio of: (a) 50/1; (b) 35/1; (c) 25/1. PBS:ethanol (1:1), 37 °C; cuvette thickness = 1 cm; the arrow indicates the direction of dialysis duration increasing.; Figure S8: The absorption spectra of ROT at different time intervals of release for PC/Chol/TPPB14 at molar ratio of: (a) 50/1; (b) 35/1; (c) 25/1. PBS:ethanol (1:1), 37 °C; cuvette thickness = 1 cm; the arrow indicates the direction of dialysis duration increasing.; Figure S9: The absorption spectra of ROT at different time intervals of release for PC/Chol/IA10(OH) at molar ratio of: (a) 50/1; (b) 35/1; (c) 25/1. PBS:ethanol (1:1), 37 °C; cuvette thickness = 1 cm; the arrow indicates the direction of dialysis duration increasing.; Figure S10: The absorption spectra of ROT at different time intervals of release for: (a) PC/Chol/IA-10(OH); (b) PC/Chol/IA-12(OH); (c) PC/Chol/IA-14(OH); (d) PC/Chol/IA-16(OH) at molar ratio of 50/1. PBS:ethanol (1:1), 37 °C; cuvette thickness = 1 cm; the arrow indicates the direction of dialysis duration increasing.; Figure S11: The absorption spectra of ROT at different time intervals of release for: (a) PC/Chol/TPPB-10; (b) PC/Chol/TPPB-12; (c) PC/Chol/TPPB-14; (d) PC/Chol/TPPB-16 at molar ratio of 50/1. PBS:ethanol (1:1), 37 °C; cuvette thickness = 1 cm; the arrow indicates the direction of dialysis duration increasing.
