# Supplementary material for: Mitochondria-Targeted Lipid Nanoparticles Loaded with Rotenone as a New Approach for the Treatment of Oncological Diseases
Source: Molecules. 2023 Oct 23;28(20):7229. doi: 10.3390/molecules28207229 (PMC10609561; doi:10.3390/molecules28207229)
Supplement: Supplementary file 1 [file molecules-28-07229-s001.zip › molecules-2628491-supplementary.pdf]

# Mitochondria-Targeted Lipid Nanoparticles Loaded with Rotenone as a New Approach for the Treatment of Oncological Diseases

Leysan Vasileva[1], Gulnara Gaynanova[1], Darya Kuznetsova[1], Farida Valeeva[1], Anna Lyubina[1], Syumbelya Amerhanova[1], Alexandra Voloshina[1], Guzel Sibgatullina[2], Dmitry Samigullin[2], Konstantin Petrov[1], Lucia Zakharova[1]*

[1]Arbuzov Institute of Organic and Physical Chemistry, FRC Kazan Scientific Center, Russian Academy of Sciences, 8 Arbuzov str., Kazan, 420088, Russia
[2]Kazan Institute of Biochemistry and Biophysics, FRC Kazan Scientific Center, Russian Academy of Sciences, 2/31 Lobachevsky str., Kazan, 420111, Russia
Correspondence: luciaz@mail.ru

**Table S1.** Extinction coefficient values of ROT in various medium, 25°C.

| Medium | ε, $M^{-1}$ $см^{-1}$ | λ, nm |
|---|---|---|
| Water | 15818 | 299 |
| Ethanol | 16606 | 295 |
| Water:Ethanol (1:1) | 18349 | 295 |
| PBS:Ethanol (1:1) | 18125 | 295 |

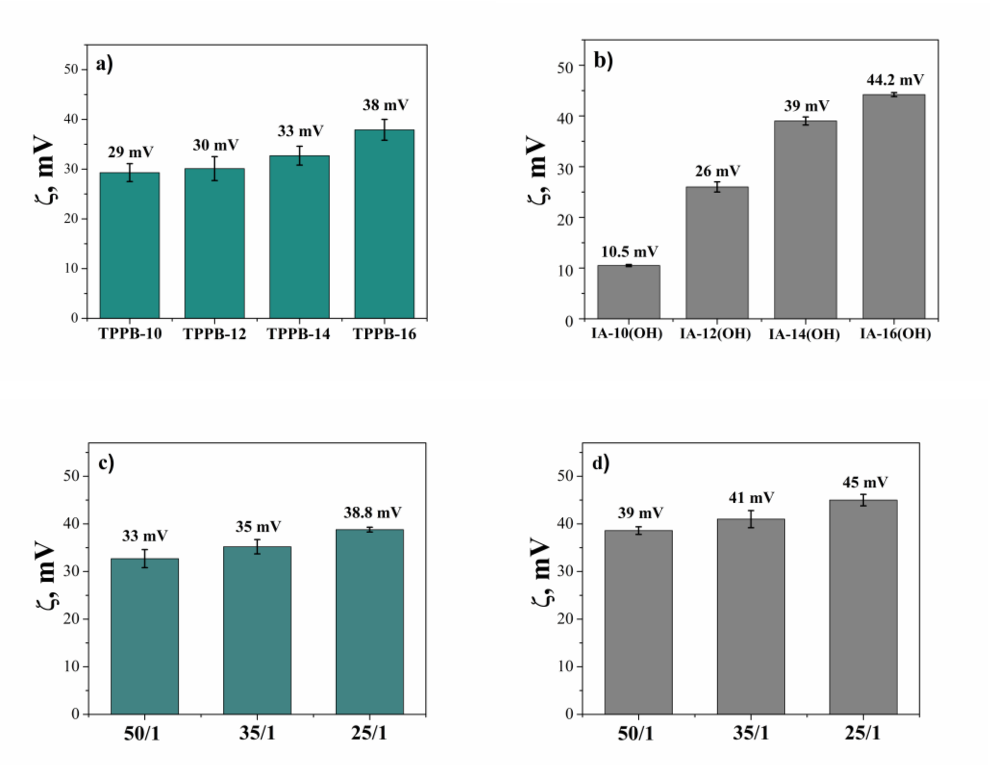


**Figure S1.** Zeta potential of PC/Chol/TPPB-n and PC/Chol/IA-n(OH) liposomes by varying: a) TPPB-n hydrocarbon tail length at a molar ratio of 50/1; b) IA-n(OH) hydrocarbon tail length at a molar ratio of 50/1; c) lipid/TPPB-14 molar ratio; d) lipid/IA-14(OH) molar ratio on the preparation day, 25°C.

**Table S2.** Cytotoxicity and SI of TPPB-n on normal and tumor cell lines.

| Surfactant | $IC_{50}$, µM | | | $SI_{Chang\ liver/HuTu\ 80}$ | $SI_{Chang\ liver/PANC-1}$ |
|---|---|---|---|---|---|
| | Tumor cell lines | | Normal cell line | | |
| | HuTu 80 | PANC-1 | Chang liver | | |
| TPPB-10 | 0.04 | 3.3 | 3.3 | 82.5 | 1 |
| TPPB-12 | 0.01 | 0.35 | 2.8 | 280 | 8 |
| TPPB-14 | 0.036 | 1.0 | 3.4 | 94.4 | 3.4 |
| TPPB-16 | 0.04 | 6.2 | 3.6 | 90 | 0.58 |

**Table S3.** Physicochemical characteristics of PC/Chol/IA-n(OH) modified liposomes (25/1) loaded with ROT (0.1 mg/mL): encapsulation efficiency (EE), hydrodynamic diameter ($D_h$), polydispersity index (PdI) and zeta potential (ζ) over time, 4°C.

| Formulation | EE, % | $D_h$, nm | PdI | ζ, mV | $D_h$, nm | PdI | ζ, mV |
|---|---|---|---|---|---|---|---|
| | | 1st day | | | 2 months | | |
| **PC/Chol/IA-10(OH)** | 95±1 | 113±0.4 | 0.109±0.005 | +15±1 | 120±1 | 0.079±0.012 | +15.1±0.5 |
| **PC/Chol/IA-12(OH)** | 95±1 | 144±1 | 0.140±0.011 | +31±2 | 119±0.1 | 0.136±0.025 | +26±3 |
| **PC/Chol/IA-14(OH)** | 94±1 | 106±0.2 | 0.181±0.023 | +43±1 | 111±1 | 0.069±0.015 | +33±3 |
| **PC/Chol/IA-16(OH)** | 93±2 | 136±2 | 0.188±0.011 | +47±1 | 118±1 | 0.068±0.014 | +38.2±0.4 |

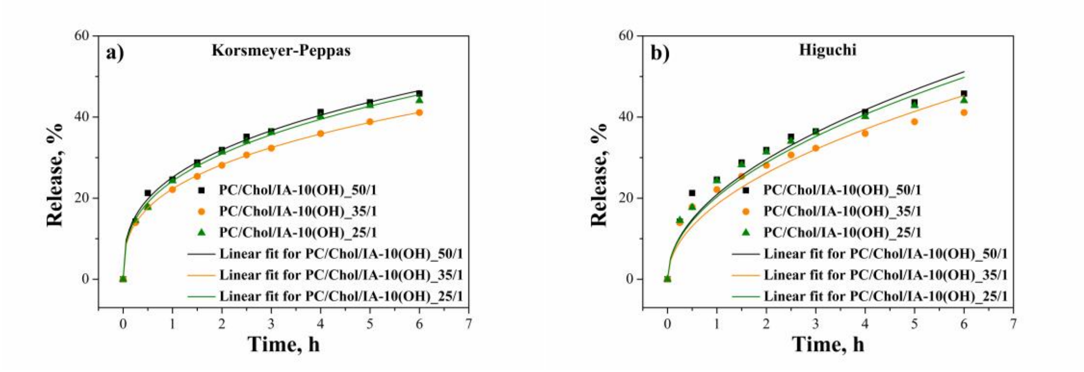


**Figure S2.** a) Korsmeyer-Peppas and b) Higuchi kinetic model fitting curves of ROT release from PC/Chol/IA-10(OH) liposomes at various molar ratio of components, 37°C.

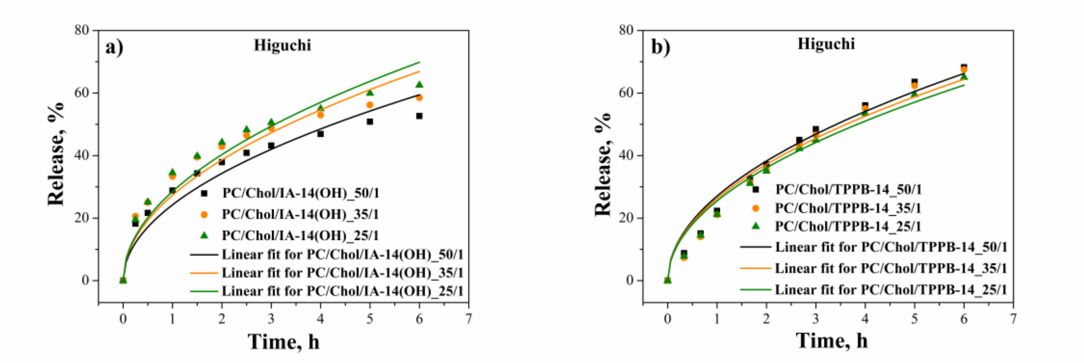

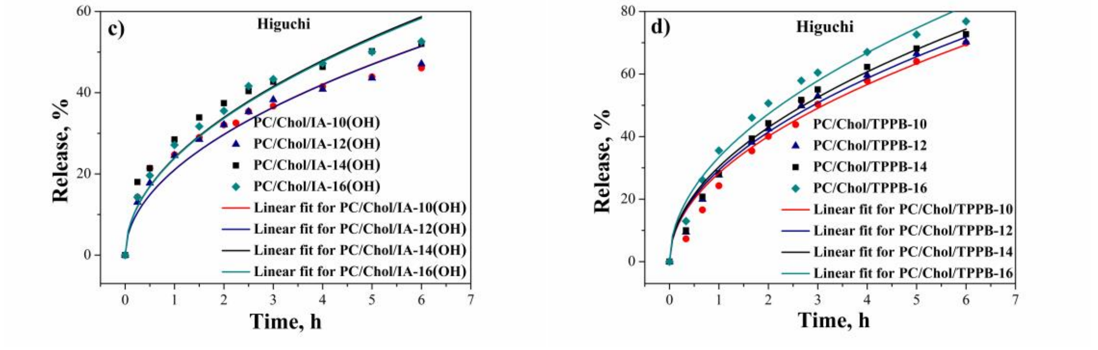


**Figure S3.** The Higuchi kinetic model fitting curves of ROT release from modified liposomes by varying: a) the molar ratio of PC/Chol/IA-14(OH); b) the molar ratio PC/Chol/TPPB-14; c) the IA-n(OH) hydrocarbon tail length at molar ratio of 50/1; d) the TPPB-n hydrocarbon tail length at molar ratio of 50/1, 37°C.

**Table S4**. The Korsmeyer-Peppas and Higuchi kinetic model fitting parameters of ROT release from PC/Chol/IA-10(OH) liposomes at various lipid/surfactant molar ratio.

| Formulation | Lipid/surfactant molar ratio | Korsmeyer-Peppas | | | Higuchi | |
|---|---|---|---|---|---|---|
| | | **k** | **n** | **$R^2$** | **k** | **$R^2$** |
| PC/Chol/IA-10(OH) | 50/1 | 25.13±0.35 | 0.344±0.011 | 0.9964 | 20.89±0.71 | 0.9317 |
| | 35/1 | 22.29±0.08 | 0.342±0.003 | 0.9998 | 18.50±0.61 | 0.9339 |
| | 25/1 | 24.20±0.33 | 0.353±0.010 | 0.9967 | 20.32±0.65 | 0.9405 |

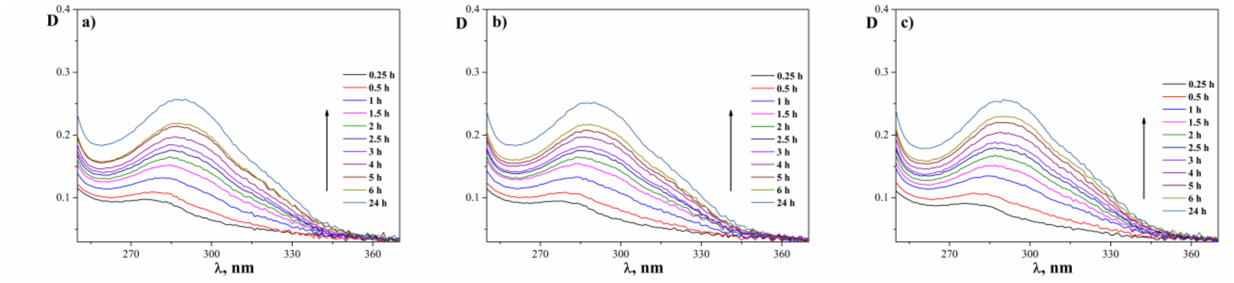


**Figure S4.** The absorption spectra of ROT at different time intervals of release for PC/Chol/IA-14(OH) at molar ratio of: a) 50/1; b) 35/1; c) 25/1. PBS:ethanol (1:1), 37°C; cuvette thickness = 1 cm; the arrow indicates the direction of dialysis duration increasing.

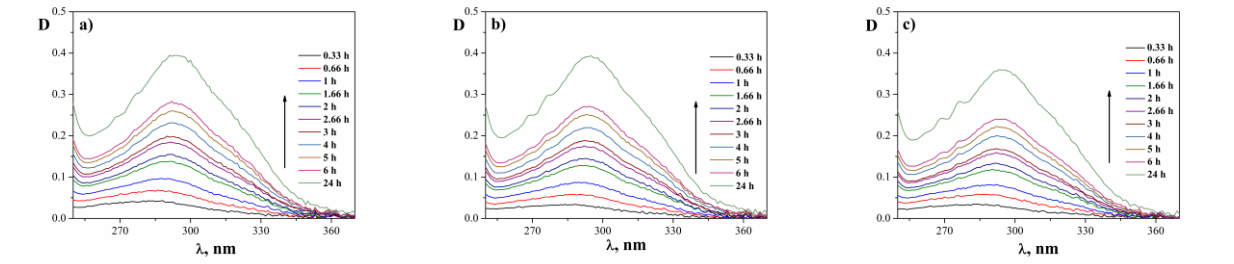

**Figure S5.** The absorption spectra of ROT at different time intervals of release for PC/Chol/TPPB-14 at molar ratio of: a) 50/1; b) 35/1; c) 25/1. PBS:ethanol (1:1), 37°C; cuvette thickness = 1 cm; the arrow indicates the direction of dialysis duration increasing.

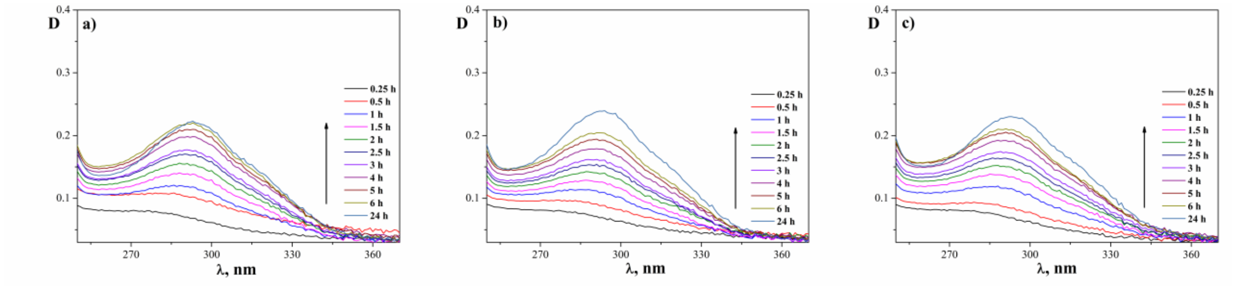


**Figure S6.** The absorption spectra of ROT at different time intervals of release for PC/Chol/IA-10(OH) at molar ratio of: a) 50/1; b) 35/1; c) 25/1. PBS:ethanol (1:1), 37°C; cuvette thickness = 1 cm; the arrow indicates the direction of dialysis duration increasing.

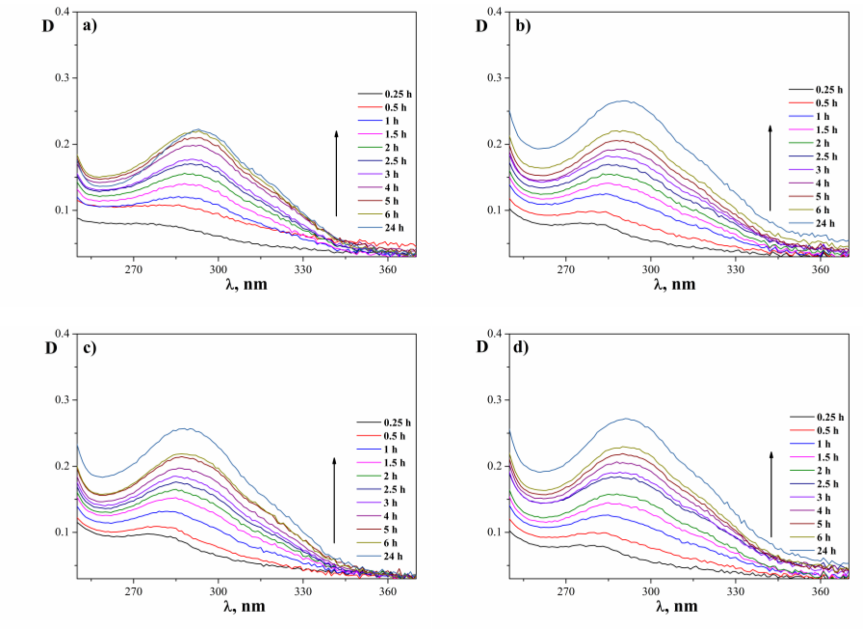


**Figure S7.** The absorption spectra of ROT at different time intervals of release for: a) PC/Chol/IA-10(OH); b) PC/Chol/IA-12(OH); c) PC/Chol/IA-14(OH); d) PC/Chol/IA-16(OH) at molar ratio of 50/1. PBS:ethanol (1:1), 37°C; cuvette thickness = 1 cm; the arrow indicates the direction of dialysis duration increasing.

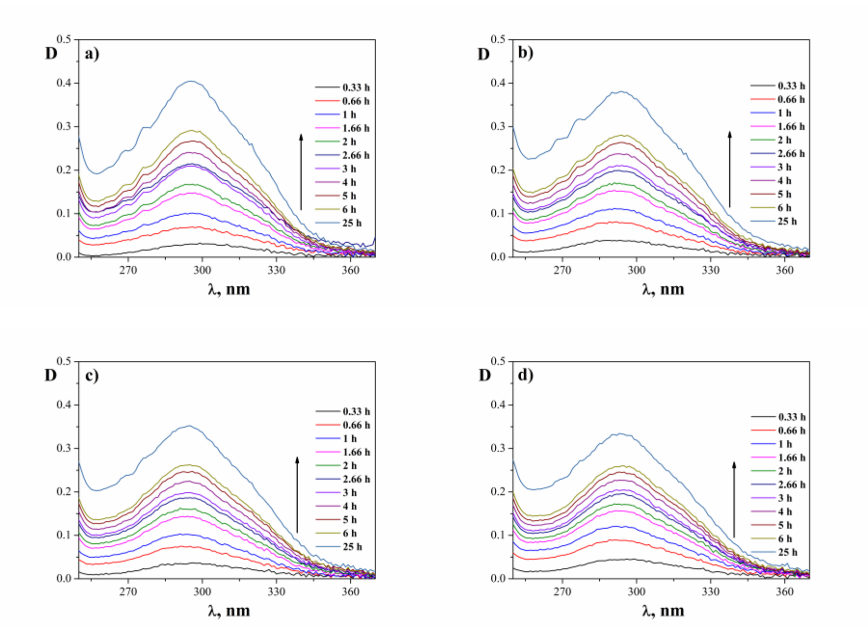


**Figure S8.** The absorption spectra of ROT at different time intervals of release for: a) PC/Chol/TPPB-10; b) PC/Chol/TPPB-12; c) PC/Chol/TPPB-14; d) PC/Chol/TPPB-16 at molar ratio of 50/1. PBS:ethanol (1:1), 37°C; cuvette thickness = 1 cm; the arrow indicates the direction of dialysis duration increasing.
